# DNA repair protein XPA is differentially expressed in colorectal cancer and predicts better prognosis

**DOI:** 10.1002/cam4.1480

**Published:** 2018-04-19

**Authors:** Xue Feng, Jingwei Liu, Yuehua Gong, Kaihua Gou, Huaiwei Yang, Yuan Yuan, Chengzhong Xing

**Affiliations:** ^1^ Tumor Etiology and Screening Department of Cancer Institute and General Surgery The First Hospital of China Medical University Shenyang 110001 China; ^2^ Liaoning Provincial Education Department Key Laboratory of Cancer Etiology and Prevention China Medical University Shenyang 110001 China

**Keywords:** Colorectal cancer, prognosis, XPA

## Abstract

As an indispensable factor in DNA damage recognition step of nucleotide excision repair, XPA interacts with a series of proteins to initiate repair process. The expression characteristics of XPA in colorectal cancer (CRC) and its influence on CRC prognosis remain elusive. Tissue specimens of CRC and nontumor adjacent tissues from 283 patients were collected. XPA protein expressions were detected by immunohistochemistry staining. Nonparametric test was used to investigate the difference of XPA expression between CRC and nontumor adjacent tissues, as well as the correlation between XPA expression and clinicopathological parameters of CRC. Univariate and multivariate Cox proportional hazards models were applied to estimate the relationship between XPA expression and CRC prognosis. Meanwhile, we analyzed TCGA data to investigate the relation between XPA mRNA expression and survival of CRC. XPA protein expression was significantly decreased in CRC tissues compared with nontumor adjacent tissues (*P* = 0.001). Subgroup analysis indicated consistently significant down‐regulation of XPA in CRC tissues in age > 60 (*P *=* *0.026), age ≤ 60 (*P* = 0.008), colon cancer (*P* = 0.009), and rectal cancer (*P* = 0.015) patients and males (*P* = 0.004). For clinicopathological parameters, CRC patients with drinking habits revealed XPA overexpression than nondrinkers (*P* = 0.032). For prognosis, CRC patients with high XPA protein expression had longer overall survival (OS) (HR = 0.62, 95%CI: 0.39–0.97, *P* = 0.037). Stratified analysis suggested a better prognosis in relation to high XPA protein expression in patients over 60 years (adjusted HR = 0.48, *P* = 0.021), with rectal cancer (HR = 0.56, *P* = 0.037), without distant metastasis (HR = 0.58, *P* = 0.033), without tumor deposits (HR = 0.40, *P* = 0.006; adjusted HR = 0.44, *P* = 0.028), and with tumor diameter over 4 cm (HR = 0.49, *P* = 0.023). DNA repair protein XPA is significantly decreased in colorectal cancer tissues than in adjacent nontumor tissues. High expression of XPA protein showed significant relationship with better survival of CRC, especially rectal cancer. XPA might be a novel biomarker but might not be an independent factor to predict prognosis of CRC patients.

## Introduction

Various human diseases, particularly cancer, mainly derive from an imbalance between DNA damage and repair 1, 2. DNA damage is induced by endogenous or exogenous stimuli 3, 4, while DNA repair is accomplished by systems including nucleotide excision repair (NER), base excision repair (BER), mismatch repair (MMR), and double‐strand break repair (DSBR) 5. NER system, which is versatile and crucial, monitors and restores multiple DNA damage of ultraviolet‐induced cyclobutane pyrimidine dimers, bulky adducts as well as DNA cross‐links 6, 7. And four key procedures participated in the NER pathway are as follows: damage recognition, damage demarcation and unwinding, damage incision, and new strand ligation 8, 9.

*Xeroderma pigmentosum group A* (*XPA*) gene, mapped to chromosome 9q22.3, includes six exons and encodes a zinc finger protein of 273 amino acids 10. As an indispensable factor in DNA damage recognition, XPA interacts with a series of NER proteins to initiate repair process 6, 11, 12. It has been revealed that cells or animals lacking XPA cannot accomplish NER 9, 13, 14, 15. Considering the critical role of XPA in NER, a number of studies have been conducted to investigate the effect of XPA on cancer 16, 17, 18. Xiang Fu et al. 19 found that high expression of XPA correlated with poor prognosis in 129 nasopharyngeal carcinoma patients treated with platinum‐based chemoradiotherapy using immunohistochemistry. In metastatic ovarian carcinoma, the results of 67 malignant effusion specimens showed that the overexpression of XPA was associated with better (progression‐free survival) PFS and (overall survival) OS 20. So far, however, the expression characteristics of XPA in CRC, which is the fourth most common cause of cancer mortality and third most frequently diagnosed cancers in both males and females in China 21, and its influence on CRC prognosis remain elusive.

In this study, we detected XPA protein expression levels in the colorectal mucosa tissues and their adjacent nontumor tissues from 283 CRC patients by immunohistochemical staining. Meanwhile, the association between XPA expression with clinicopathological parameters and prognosis in CRC patients was analyzed to clarify the latent effect of XPA on the progression and prognosis of CRC.

## Materials and Methods

### Patients and tissue specimens

The study was approved by the Institute Research Medical Ethics Committee of the First Affiliated Hospital of China Medical University, and written informed consents were obtained from all individuals. Patients were enrolled from the First Affiliated Hospital of China Medical University who experienced surgical operation between October 2012 and July 2015. Tissue specimens including 283 CRC tissues and the corresponding nontumor adjacent tissues were collected in our study.

On the basis of the World Health Organization criteria, the tissue samples of CRC diagnosed on the account of histological results. International Union Against Cancer (UICC)/American Joint Committee on Cancer (AJCC) (seventh edition, 2010) was used to confirm TNM staging of CRC in the following of postoperative pathological diagnosis. Three criteria were made to exclude CRC patients (1) having XP disease, (2) accepting preoperative chemotherapy or radiation, and (3) having hereditary nonpolyposis colorectal cancer (HNPCC). The follow‐up was performed until August 2017. A total of 266 cases were included to analyze the prognosis (mean survival time was 37.9 months; the time of follow‐up ranged from 1 month to 56 months; 79 of them died), while the rest 17 cases were not included for the OS analysis because loss of follow‐up. The study defines overall survival (OS) as the period from the date of operation to death. The patients who smoke at least one cigarette daily for at least 1 year were regarded as the cases with history of smoking. Meanwhile, the study defines history of drinking as the mean alcohol ingest per day for at least 50 g and lasting for at least 1 year.

### Immunohistochemistry

Immunohistochemistry was performed mainly as previously described 22. Tissues, which were fixed with formalin and embedded with paraffin, were cut into 4‐*μ*m‐thick sections and mounted in a poly‐l‐lysine‐coated glass slides. After routine deparaffinization, rehydration in a graded alcohol series and washing in tap water, the tissue sections were exposed to 10 mmol/L citrate buffer (PH 6.0) for 90 sec in a steam pressure cooker for antigen retrieval. Endogenous peroxidase was blocked using 3% hydrogen peroxide for 10 min, and then, the tissue sections were washed with phosphate‐buffered saline (PBS, PH 7.4). To lessen the nonspecific binding, 10% normal goat serum was subsequently used to block tissue collagen for 10 min. The mouse monoclonal antibody anti‐XPA (ab‐2352, 1:200 dilution; Abcam, Cambridge, U.K.) was used as the primary antibody to detect XPA protein expression and incubated for 60 min at room temperature (24–27°C). After that, the sections were rinsed by PBS for 10 min each and then incubated with biotinylated secondary antibody (goat anti‐rabbit antibody, Maixin Inc., Fujian, China) and streptavidin–biotin–peroxidase for 10 min each at temperature (24–27°C). Slides were stained with DAB (DAB‐0031, Maixin Inc., Fujian, China) chromogenic reagent for 80 sec. At last, the slides were dehydrated and fixed by resin. Meanwhile, we used three ways to control the quality of IHC. First, we used negative (PBS was used to substitute primary and secondary antibodies, respectively) and positive controls in the IHC staining to avoid false‐negative or false‐positive results. Second, the DAB staining was observed by microscope in case that the staining was overestimated or underestimated. Third, two pathologists independently scored the XPA expression level in a double‐blind manner.

### Evaluation of immunohistochemistry

XPA protein expressions in the different tissues were read and scored independently by two pathologists, in accordance with the double‐blind principle. On the basis of immunohistochemistry semiquantification method, the pathologists evaluated the area and intensity of the staining results. And if the differences between the results of the pathologists were more than one grade, more scopes would be selected and the final scores would be discussed and concluded by the two pathologists. Semiquantitative scoring criterion was used to evaluate the expression of XPA in nucleus. The staining intensity of cancer cells was graded on a scale of 0–3(I_0_–I_3_): I_0_ (no staining), I_1_ (light brown), I_2_ (brown staining), and I_3_ (heavy brown staining) (intermediary intensity between two levels was defined as I_0.5_, I_1.5_, and I_2.5)_; the proportion of stained cells were recorded as (P_0_–P_3_): 0–5% (P_0_), 6–25% (P_1_), 26–50% (P_2_), 51–75% (P_3_), and 76–100% (4). The final IS scores were accumulated by the formula: IS score = I_n_ × P_m_. At last, the XPA protein expression was graded as follows: negative (–), score = 0; weak expression (+), score = 0.5–4; moderate expression (++), score = 4.5–8; and strong expression (+++), score = 9–12. As the median for immunohistochemistry score, score 4.5 was selected as the cutoff value to distinguish high or low expression for XPA protein.

### Obtainment of data from TCGA database

The Cancer Genome Atlas (TCGA) is a publicly available database that has generated comprehensive, multidimensional maps of the important genomic changes in 33 types of cancer. In this study, data of 478 colon adenocarcinoma cases (TCGA‐COAD, provisional) with expression and clinicopathological information were downloaded. Additionally, data of 166 rectum adenocarcinoma cases (TCGA‐READ, provisional) were obtained to analyze the relationship of XPA mRNA expression with CRC prognosis.

### Statistical analysis

All statistical analyses were performed using SPSS software, Chicago, IL (version 18.0). The comparison of XPA expression between CRC and nontumor adjacent tissues was assessed by nonparametric test. The correlation between XPA expression and clinicopathological parameters of CRC was also conducted by nonparametric test. The study applied Kaplan–Meier method to visualize the patient survival time and employed log‐rank tests to analyze the difference between groups. Univariate Cox proportional hazards model was applied to estimate the relationship between the expression of XPA and CRC prognosis, and multivariate Cox proportional hazards model was used to evaluate the association adjusted by age, gender, TNM stage, and differentiation degree. *P* values <0.05 were considered statistically significant.

## Results

### Baseline characteristics

The baseline characteristics of the 283 CRC patients included are shown in Table 1. Altogether, 165 males and 118 females were enrolled with a median survival time (MST) of 44.55 months and 47.14 months, respectively. Totally, 152 CRC patients were over 60 years of age, while 131 cases were younger than 60. The location of colorectal cancer included colon (80 cases) and rectum (202 cases). TNM staging was as follows: stage I, 73; stage II, 69; stage III, 121; and stage IV, 20.

**Table 1:** Clinicopathological parameters and survival in CRC

Characteristics	CRC	Cases of events	MST	*P*
Gender				
Male	165	52	44.55	0.252
Female	118	30	47.14	
Age (years)				
>60	152	50	43.64	0.171
≤60	131	32	47.25	
Smoking				
Yes	72	19	46.66	0.578
No	209^a^	63	45.19	
Drinking				
Yes	31^a^	8	46.03	0.615
No	237	72	45.18	
Tumor location				
Colon	80	24	44.41	0.889
Rectum	202^a^	57	46.29	
TNM stage				
I	73	14	26.62	<0.001
II	69	50	41.32	
III	121	12	49.72	
IV	20	6	53.35	
Invasive extent				
T1–2	86	11	52.04	<0.001
T3–4	197	71	42.77	
Lymph node metastasis				
Positive	135	62	39.29	<0.001
Negative	148	20	51.26	
Distant metastasis				
Positive	20	14	26.62	<0.001
Negative	263	68	46.98	
Tumor deposit				
Positive	31	17	29.16	<0.001
Negative	184^a^	44	44.49	
Perineural invasion				
Positive	148	49	39.75	0.004
Negative	71^a^	13	49.13	
Vessel carcinoma embolus				
Positive	65	27	38.92	0.006
Negative	218	55	47.20	
Growth pattern				
Infiltrative	163^a^	61	42.07	<0.001
Nested/cloddy	119	21	50.28	
Differentiation degree				
Poor/mucinous	79	39	36.41	<0.001
Well/moderate	191^a^	37	49.98	
Maximum diameter(cm)				
>4	133	45	42.92	0.036
≤4	149^a^	36	48.10	
Family history				
Positive	57	15	45.65	0.478
Negative	226	67	45.43	
Chemotherapy				
Yes	107^a^	27	48.14	0.409
No	111	30	44.53	

CRC, colorectal cancer; MST, median survival time.

a Incomplete information.

### Down‐regulation of XPA in CRC tissues than nontumor adjacent tissues

**Figure 1:**
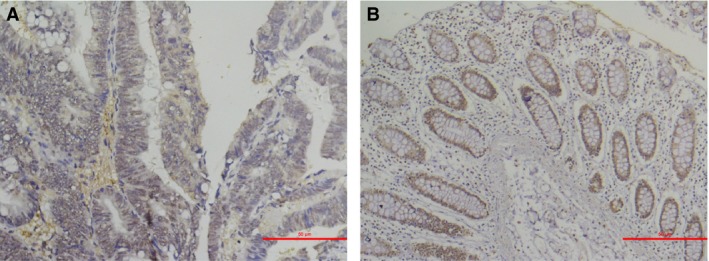
Representative photomicrographs of immunohistochemical staining of XPA in CRC specimens and adjacent nontumor specimens. (A) Colorectal cancer tissues and (B) adjacent nontumor tissues of CRC. Original magnification, ×200.

**Figure 2:**
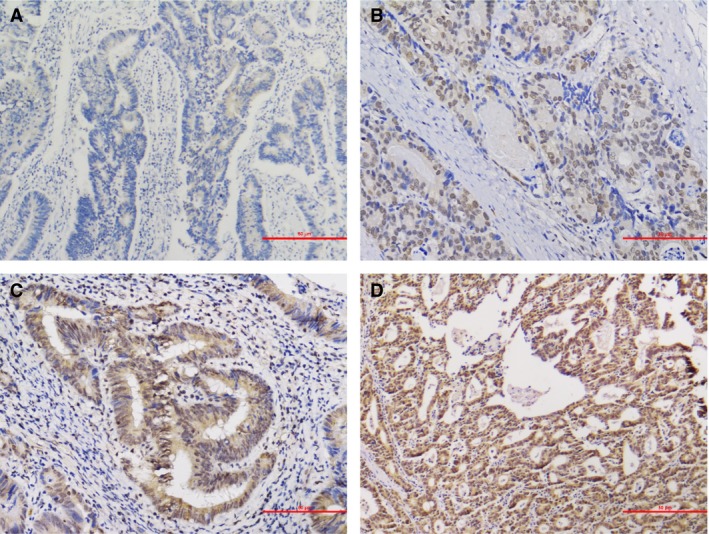
Different XPA expression levels in CRC tissues. (A) negative (−), (B) weakly positive (+), (C) moderately positive (++), and (D) strongly positive (+++). Magnification, ×200.

**Figure 3:**
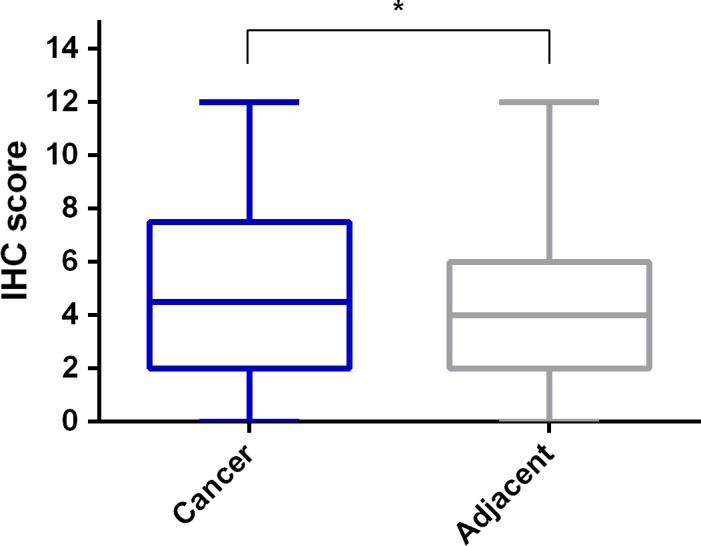
XPA protein expression was significantly decreased in CRC tissues compared with nontumor adjacent tissues. According to the Mann–Whitney U‐test, the differential expression of XPA between CRC specimens and nontumor adjacent specimens was visualized by scatter plots. *: *P*<0.05

The representative immunohistochemistry staining of CRC tissue and nontumor adjacent tissue is shown in Figure 1 (Figure 1A and B), respectively. Figure 2 demonstrated four different staining grades as negative (−), light positive (+), positive (++), and strong positive (+++). The detailed results of the expression profile of XPA in CRC and nontumor adjacent tissues are summarized in Table 2. According to the Mann–Whitney U‐test, XPA protein expression was significantly decreased in CRC tissues compared with nontumor adjacent tissues (*P* = 0.001), which is visualized by scatter plots in Figure 3.

**Table 2:** XPA expression in CRC and nontumor adjacent tissues

Category	Group	Cases	(−)	(+)	(++)	(+++)	PR (%)	*P*
			*n* (%)	*n* (%)	*n* (%)	*n* (%)		
Overall	CRC	275	11 (4.0)	120 (43.6)	108 (39.3)	36 (13.1)	96.0	**0.001**
	Adjacent	275	27 (9.8)	64 (23.3)	123 (44.7)	61 (22.2)	90.2	
Male	CRC	161	7 (4.3)	72 (44.7)	59 (36.6)	23 (14.3)	95.7	**0.004**
	Adjacent	161	17 (10.6)	36 (22.4)	70 (43.5)	38 (23.6)	89.4	
Female	CRC	114	4 (3.5)	48 (42.1)	49 (43.0)	13 (11.4)	96.5	0.067
	Adjacent	114	10 (8.8)	28 (24.6)	53 (46.5)	23 (20.2)	91.2	
≤60	CRC	130	5 (3.8)	65 (50.0)	43 (33.1)	17 (13.1)	96.2	**0.008**
	Adjacent	130	14 (10.8)	29 (22.3)	63 (48.5	24 (18.5)	89.2	
>60	CRC	145	6 (4.1)	55 (37.9)	65 (44.8)	19 (13.1)	95.9	**0.026**
	Adjacent	145	13 (9.0)	35 (24.1)	60 (41.4)	37 (25.5)	91.0	
Colon	CRC	78	2 (2.6)	38 (48.7)	29 (37.2)	9 (11.5)	97.4	**0.009**
	Adjacent	78	9 (11.5)	15 (19.2)	38 (48.7)	16 (20.5)	88.5	
Rectum	CRC	196	9 (4.6)	82 (41.8)	78 (39.8)	27 (13.8)	95.4	**0.015**
	Adjacent	196	18 (9.2)	48 (24.5)	85 (43.4)	45 (23.0)	90.8	

PR, positive rate. Negative (−), light positive (+), positive (++), strong positive (+++) staining. Mann–Whitney U‐test of nonparametric test to compare the XPA protein expression between CRC and adjacent tissues.

The bold values: *P*<0.05

Subgroup analysis based on age and tumor location suggested consistently significant down‐regulation of XPA in CRC tissues than in their adjacent tissues in age > 60 (*P* = 0.026), age ≤ 60 (*P* = 0.008), colon cancer (*P* = 0.009), and rectal cancer (*P* = 0.015). In addition, male patients showed low XPA expression in CRC tissues compared with adjacent tissues (*P* = 0.004), but no significant difference was observed in female individuals (*P* = 0.067).

### Association between XPA protein expression and clinicopathological parameters of CRC patients

CRC patients were stratified according to variables including gender, age, smoking, drinking, TNM stage, and tumor invasion depth, and Mann–Whitney U‐test was performed to explore the differential expression of XPA between groups (Table 3). The results indicated that XPA protein expression correlated with drinking status: CRC patients with drinking habits revealed XPA overexpression than nondrinkers (*P* = 0.032). However, most comparisons of other clinicopathological parameters of CRC did not demonstrate significant difference (*P* > 0.05).

**Table 3:** Association between XPA expression and clinicopathological parameters in CRC

Variables	Cases	(−)	(+)	(++)	(+++)	PR (%)	*P*
		*n*	*n*	*n*	*n*		
Gender							
Male	165	8 (4.8)	73 (44.2)	61 (37.0)	23 (13.9)	95.2	0.734
Female	118	5 (4.2)	50 (42.4)	50 (42.4)	13 (11.0)	95.8	
Age (years)							
>60	152	8 (5.3)	57 (37.5)	68 (44.7)	19 (12.5)	94.7	0.218
≤60	131	5 (3.8)	66 (43.4)	43 (28.3)	17 (13.0)	96.2	
Smoking							
Yes	72	2 (2.8)	7 (9.7)	31 (20.4)	12 (16.7)	97.2	0.102
No	209^a^	11 (2.1)	95 (45.5)	80 (38.3)	23 (11.0)	94.7	
Drinking							
Yes	31^a^	1 (3.2)	9 (29.0)	14 (45.2)	7 (22.6)	96.8	0.032
No	237	12 (5.1)	108 (45.6)	92 (38.8)	25 (10.5)	94.9	
Tumor location							
Colon	80	2 (2.5)	39 (48.8)	30 (37.5)	9 (11.3)	97.5	0.372
Rectum	202^a^	11 (5.4)	84 (41.6)	80 (39.6)	27 (13.4)	94.6	
TNM stage							
I	73	2 (2.7)	33 (45.2)	30 (31.1)	8 (11.0)	97.3	0.863
II	69	5 (7.2)	25 (36.2)	31 (44.9)	8 (11.6)	92.8	
III	121	4 (43.3)	58 (47.9)	44 (36.4)	15 (12.4)	96.7	
IV	20	2 (10.0)	7 (35.0)	6 (30.0)	5 (25.0)	90.0	
Invasive depth							
T1–2	86	2 (2.3)	38 (44.2)	37 (43.0)	9 (10.5)	97.7	0.653
T3–4	197	11 (5.6)	85 (43.1)	74 (37.6)	27 (13.7)	94.4	
Lymph node metastasis							
Positive	135	6 (4.4)	64 (47.4)	48 (35.6)	17 (12.6)	95.6	0.552
Negative	148	7 (4.7)	59 (39.9)	63 (42.6)	19 (12.8)	95.3	
Distant metastasis							
Positive	20	2 (10.0)	7 (35.0)	6 (30.0)	5 (25.0)	90.0	0.997
Negative	263	11 (4.2)	116 (44.1)	105 (39.9)	31 (11.8)	95.8	
Tumor deposits							
Positive	31	1 (3.2)	11 (35.5)	13 (41.9)	6 (19.4)	96.8	0.098
Negative	184^a^	11 (6.0)	81 (44.0)	73 (39.7)	19 (10.3)	94.0	
Perineural invasion							
Positive	148	10 (6.8)	67 (45.3)	53 (35.8)	18 (12.2)	93.2	0.146
Negative	71^a^	2 (2.8)	28 (39.4)	33 (46.5)	8 (11.3)	97.2	
Lymphatic/venous invasion							
Positive	65	3 (4.6)	27 (41.5)	25 (38.5)	10 (15.4)	95.4	0.729
Negative	218	10 (4.6)	96 (44.0)	86 (39.4)	26 (11.9)	95.4	
Growth pattern							
Infiltrative	163^a^	11 (6.7)	74 (45.4)	59 (36.2)	19 (11.7)	93.3	0.085
Cloddy/nested	119	2 (1.7)	49 (41.2)	51 (42.9)	17 (14.3)	98.3	
Differentiation degree							
Poor/mucinous	79	7 (8.9)	35 (44.3)	30 (38.0)	7 (8.9)	91.1	0.332
Well/moderate	191^a^	6 (3.1)	83 (43.5)	76 (39.8)	26 (13.6)	96.9	
Maximum diameter (cm)							
>4	133	8 (6.0)	53 (39.8)	54 (40.6)	18 (13.5)	94.0	0.521
≤4	149^a^	5 (3.4)	69 (46.3)	57 (38.3)	18 (12.1)	96.6	
Family history							
Positive	57	2 (3.5)	26 (45.6)	21 (36.8)	8 (14.0)	96.5	0.911
Negative	226	11 (4.9)	97 (42.9)	90 (39.8)	28 (12.4)	95.1	

PR, positive rate. Negative (−), light positive (+), positive (++), strong positive (+++) staining.

The association of XPA expression with TNM stage was analyzed by Kruskal–Wallis H‐test of nonparametric test. For other clinicopathological parameters, Mann–Whitney U‐test of nonparametric test was used.

a Incomplete information.

The bold values: P<0.05

### Relationship between XPA expression and CRC prognosis

**Figure 4:**
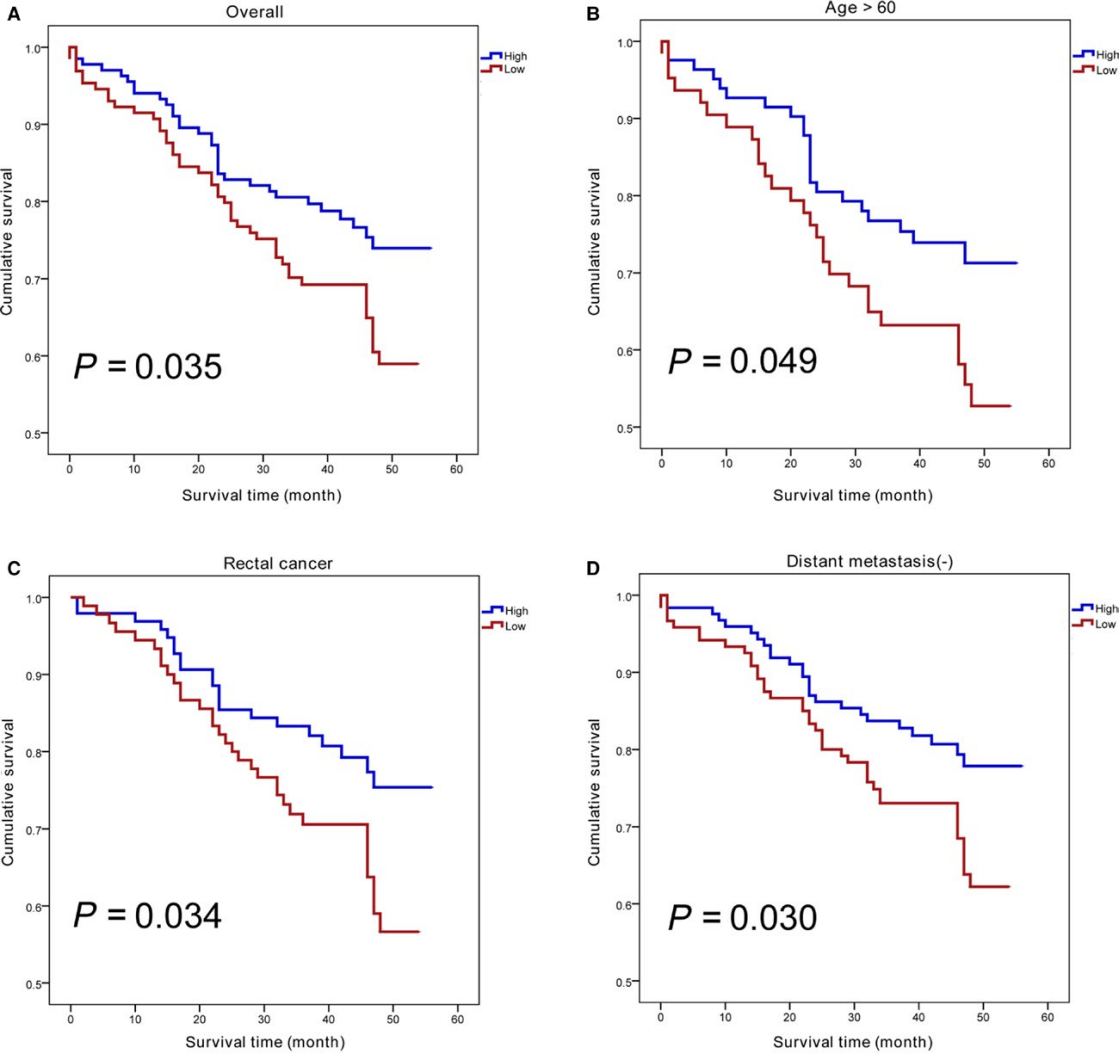
High expression of XPA correlates with the prognosis in CRC patients. (A) Kaplan–Meier analysis and log‐rank test for overall survival according to XPA expression level; (B) patients over 60 years of age with high XPA expression exhibited longer survival time than those with low XPA expression; (C) rectal cancer individuals who expressed higher XPA protein demonstrated favorable prognosis; (D) subgroup without distant metastasis also identified XPA expression as a good indicator for CRC prognosis.

The cutoff value of IS was 4.5 in this study as it was the median score for immunohistochemistry staining of XPA in CRC (IS ≥ 4.5 means high expression, and IS < 4.5 means low expression). To investigate whether XPA protein expression could indicate CRC prognosis, Cox proportional hazards model was applied to estimate the relationship between the expression of XPA and CRC survival (Table 4). Univariate Cox proportional hazards model revealed that CRC patients with high XPA protein expression had longer overall survival (OS) (HR = 0.62, 95%CI: 0.39–0.97, *P* = 0.037, Fig. 4A). Multivariate Cox proportional hazards model adjusting for age, gender, TNM stage, and differentiation degree did not show significant relation with CRC survival (adjusted HR = 0.68, 95% CI: 0.42–1.09, *P* = 0.107).

**Table 4:** Correlation between XPA expression and survival in CRC

	Cases	Cases of events	MST	Univariate			Multivariate		
				HR	95% CI	*P*	HR	95% CI	*P*
XPA expression									
Low (IS < 4.5)	129	46	42.25						
High (IS ≥ 4.5)	134	32	47.52	0.62	0.39–0.97	**0.037**	0.68	0.42–1.09	0.107
Stratification									
Age									
>60									
Low	63	27	39.76						
High	82	22	45.74	0.58	0.33–1.01	0.055	0.48	0.26–0.89	**0.021**
≤60									
Low	66	19	44.63						
High	52	10	49.23	0.59	0.28–1.28	0.183	0.81	0.38–1.77	0.601
Location									
Rectum									
Low	90	32	43.06						
High	96	21	48.54	0.56	0.32–0.97	**0.037**	0.59	0.33–1.05	0.072
Colon									
Low	39	14	40.24						
High	37	10	45.20	0.70	0.31–1.57	0.390	0.85	0.36–2.02	0.710
Distant metastasis									
Positive									
Low	9	7	20.44						
High	11	7	27.18	0.58	0.19–1.74	0.330	0.71	0.19–2.72	0.618
Negative									
Low	120	25	43.66						
High	123	39	49.02	0.58	0.35–0.96	**0.033**	0.61	0.36–1.04	0.072
Tumor deposits									
Positive									
Low	10	6	27.90						
High	19	10	28.47	0.89	0.32–2.45	0.823	1.22	0.43–3.47	0.711
Negative									
Low	88	29	41.34						
High	85	13	46.72	0.40	0.21–0.77	**0.006**	0.44	0.21–0.92	**0.028**
Max diameter (cm)									
>4									
Low	58	27	37.37						
High	61	16	45.91	0.49	0.26–0.91	**0.023**	0.62	0.32–1.18	0.143
≤4									
Low	70	18	46.67						
High	73	16	48.29	0.83	0.42–1.63	0.589	0.82	0.39–1.70	0.586

CI, confidence interval; HR, hazard radio; MST, median survival time. IS, the immunohistochemistry score.

The bold values: P<0.05

Stratified analysis based on age and tumor location suggested that patients over 60 years of age with high XPA expression exhibited longer survival time than those with low XPA expression (adjusted HR = 0.48, 95% CI: 0.26–0.89, *P* = 0.021, Fig. 4B); rectal cancer individuals who expressed higher XPA protein demonstrated favorable prognosis (HR = 0.56, 95% CI: 0.32–0.97, *P* = 0.037, Fig. 4C). In the subgroup without distant metastasis, high XPA expression showed significant association with better OS (HR = 0.58, 95% CI: 0.35–0.96, *P* = 0.033, Fig. 4D). Both univariate and multivariate analyses indicated significant correlation between high XPA expression and decreased hazards of death in the CRC cases without tumor deposits (HR = 0.40, 95% CI: 0.21–0.77, *P* = 0.006, adjusted HR = 0.44, 95% CI: 0.21–0.92, *P* = 0.028). Besides, the subgroup with tumor diameter over 4 cm also identified XPA expression as a good indicator for CRC prognosis (HR = 0.49, 95% CI: 0.26–0.91, *P* = 0.023). However, no significant relation was observed according to the subgroup analysis of TNM stage, invasion depth, lymph node metastasis, growth pattern, differentiation degree, and chemotherapy after initial surgical operation (Table S1). According to the TCGA results, the association between XPA mRNA expression and survival of CRC was not statistically significant (Table S2).

## Discussion

XPA, containing a zinc‐finger domain, displays a damaged DNA‐binding activity, which is essential for assembly of the preincision complex during nucleotide excision repair 11, 23. It has been reported that XPA exerts regulatory role not only by recognizing the existence of DNA damage, but, along with its interaction partner RPA, also in monitoring proper three‐dimensional arrangement of NER complex ahead of activation of endonuclease subunits 3, 24, 25, 26. Considering its important function in NER pathway, XPA is probably implicated in diseases related to imbalance between DNA damage and repair. However, the specific role of XPA in the progression and prognosis in CRC was still ambiguous. In this study including 283 CRC patients in China, we, for the first time, elucidated that DNA repair protein XPA is significantly decreased in colorectal cancer tissues than adjacent nontumor tissues. Univariate Cox proportional hazards model revealed that CRC patients with high XPA protein expression had longer overall survival (OS), but the association was not statistically significant in multivariate analysis. Besides, no significant relation was observed between XPA mRNA expression and survival of CRC according to TCGA results. According to the results of multivariate analysis and TCGA data, we suggested that XPA might be a promising biomarker but might not be an independent factor to predict prognosis of CRC patients.

In the present study, differential expression of XPA between colorectal cancer and nontumor adjacent tissues was explored. We found that XPA protein expression was significantly decreased in CRC tissues compared with nontumor adjacent tissues. Subgroup analysis suggested consistently significant difference in age over 60 years, age less than 60 years, colon cancer, rectal cancer, and males except that female individuals showed borderline significance (*P* = 0.067). These consistent findings ensure the phenomenon of decreased XPA expression in CRC tissues than in adjacent normal tissues, regardless of other factors. Previous studies on other types of cancer also came out with similar results: One research investigated twenty DNA repair pathway genes in 52 Dukes’ C colorectal cancer in Americans and revealed that only XPA had a lower RNA level in tumor samples than in matched normal ones 27; another study in Italians found significantly lower transcriptional expression of XPA in 50 nonsmall cell lung cancer (NSCLC) specimens compared with normal matched samples 28; bladder cancer also expressed low XPA of both mRNA and protein levels than nontumor bladder tissue, which was closely related to chromosomal aberrations 29. Taken together, the above‐mentioned results from other types of cancer could, at least in part, confirm our findings of XPA down‐regulation in CRC.

The association of XPA protein expression with the overall survival of CRC was also explored in this study. After classifying the CRC patients into high and low XPA expression groups by immunohistochemistry scores, we revealed a significantly increased survival time of individuals with high XPA protein expression. As for tumor location, the relationship was more obvious in rectal cancer rather than in colon cancer. Previously, the predictive role of high XPA expression for better prognosis has also been found in other types of cancers other than CRC: An Italian study investigated 171 ovarian cancer cases and suggested a longer OS and progression‐free survival (PFS) in cases that overexpressed XPA mRNA; similarly, high XPA protein expression in ovarian cancer has been regarded as an indicator for favorable prognosis according to a Norwegian research 20; Hyo Jung Cho et al. 30 found in 50 liver cancer cases in Korean population that low XPA mRNA level confers to worse survival. From this point of view, the correlation of up‐regulation of XPA with increased survival time might be applicable to not only CRC but also other types of cancers, the molecular mechanism of which requires further investigations to elucidate. Additionally, CRC patient subgroups without distant metastasis, without tumor deposits, or with tumor diameter over 4 cm demonstrated a more significant relationship with better overall survival. Thus, the influence of certain clinicopathological parameters on the implication of XPA in CRC progression is an intriguing direction for future researches.

The observations of differential expression of XPA in CRC and its predictive potential for overall survival enlighten our understanding of the complex participation of NER in the development and progression of CRC. Considering the core position of XPA in NER pathway, we assumed that the down‐regulation of XPA in CRC tissues might arise from the impairment of NER capacity upon colorectal carcinogenesis and the low XPA protein expression, which indicates degraded nuclear expression repair in CRC patients, might help create poor prognosis. On the contrary, sufficient NER ability did not benefit cancer patients from the aspect of chemotherapy, because platinum‐based chemotherapeutic regimens destroy cancer cells mainly via DNA damage. As the one of the toughest challenges for cancer treatment, chemotherapeutic resistance for platinum has been detected in XPA‐overexpressed nasopharyngeal cancer 19. Whether XPA contributes to CRC chemotherapeutic resistance remains to be clarified in the future. Biomarkers that could predict survival of cancer patients are urgently in need for clinical doctors to make individualized treatment plans and follow‐up management. In this study, the cutoff value (4.5) we used was based on our group of patients. More reliable cutoff value should be explored by multiple investigations based on different ethnicities. The obvious relation between XPA protein overexpression and favorable CRC prognosis in our study might provide useful clues for elucidating colorectal development, offering novel idea for effective treatment and improving survival.

## Conclusion

In summary, DNA repair protein XPA is significantly decreased in colorectal cancer tissues than adjacent nontumor tissues. High expression of XPA protein showed significant relationship with better survival of CRC, especially rectal cancer. XPA might be a novel biomarker but might not be an independent factor to predict prognosis of CRC patients.

## Conflict of Interest

All authors declare that there is no conflict of interest.

## Supporting information


**Table S1.** Correlation between XPA expression and survival in CRC.

**Table S2.** Correlation between XPA mRNA expression and survival in colon cancer and rectal cancer ( based on TCGA).
