# Supplementary material for: DNA repair protein XPA is differentially expressed in colorectal cancer and predicts better prognosis
Source: Cancer Med. 2018 Apr 19;7(6):2339–49. doi: 10.1002/cam4.1480 (PMC6010851; doi:10.1002/cam4.1480)
Supplement: Supplementary file 1 — Table S1. Correlation between XPA expression and survival in CRC. [file CAM4-7-2339-s001.docx]

Supplementary Table 1.Correlation between XPA expression and survival in CRC.

| **XPA expression** | **Cases** | **Events** | **MSTa** |  | **Univariate** |  |  |  | **Multivariate** |  |
| --- | --- | --- | --- | --- | --- | --- | --- | --- | --- | --- |
|  |  |  |  | **HR** | **95%CI** | **P** |  | **HR** | **95%CI** | **P** |
| Low(IS＜4.5) | 129 | 46 | 42.25 |  |  |  |  |  |  |  |
| High(IS≥4.5) | 134 | 32 | 47.52 | 0.62 | 0.39-0.97 | **0.037** |  | 0.68 | 0.42-1.09 | 0.107 |
| **Stratification** |  |  |  |  |  |  |  |  |  |  |
| **Gender** |  |  |  |  |  |  |  |  |  |  |
| **Male** |  |  |  |  |  |  |  |  |  |  |
| Low(IS＜4.5) | 78 | 28 | 41.53 |  |  |  |  |  |  |  |
| High(IS≥4.5) | 76 | 20 | 46.78 | 0.68 | 0.38-1.21 | 0.187 |  | 0.84 | 0.46-1.51 | 0.554 |
| **Female** |  |  |  |  |  |  |  |  |  |  |
| Low(IS＜4.5) | 51 | 18 | 43.33 |  |  |  |  |  |  |  |
| High(IS≥4.5) | 58 | 12 | 47.69 | 0.54 | 0.26-1.12 | 0.100 |  | 0.46 | 0.21-1.02 | 0.056 |
| **Age** |  |  |  |  |  |  |  |  |  |  |
| ＞60 |  |  |  |  |  |  |  |  |  |  |
| Low(IS＜4.5) | 63 | 27 | 39.76 |  |  |  |  |  |  |  |
| High(IS≥4.5) | 82 | 22 | 45.74 | 0.58 | 0.33-1.01 | **0.055** |  | 0.48 | 0.26-0.89 | **0.021** |
| ＜60 |  |  |  |  |  |  |  |  |  |  |
| Low(IS＜4.5) | 66 | 19 | 44.63 |  |  |  |  |  |  |  |
| High(IS≥4.5) | 52 | 10 | 49.23 | 0.59 | 0.28-1.28 | 0.183 |  | 0.81 | 0.38-1.77 | 0.601 |
| **Location** |  |  |  |  |  |  |  |  |  |  |
| **Rectum** |  |  |  |  |  |  |  |  |  |  |
| Low(IS＜4.5) | 90 | 32 | 43.06 |  |  |  |  |  |  |  |
| High(IS≥4.5) | 96 | 21 | 48.54 | 0.56 | 0.32-0.97 | **0.037** |  | 0.59 | 0.33-1.05 | 0.072 |
| **Colon** |  |  |  |  |  |  |  |  |  |  |
| Low(IS＜4.5) | 39 | 14 | 40.24 |  |  |  |  |  |  |  |
| High(IS≥4.5) | 37 | 10 | 45.20 | 0.70 | 0.31-1.57 | 0.390 |  | 0.85 | 0.36-2.02 | 0.710 |
| **TNM stage** |  |  |  |  |  |  |  |  |  |  |
| Ⅰ-Ⅱ |  |  |  |  |  |  |  |  |  |  |
| Low(IS＜4.5) | 62 | 9 | 49.01 |  |  |  |  |  |  |  |
| High(IS≥4.5) | 72 | 8 | 52.63 | 0.71 | 0.27-1.84 | 0.481 |  | 0.79 | 0.28-2.19 | 0.640 |
| Ⅲ-Ⅳ |  |  |  |  |  |  |  |  |  |  |
| Low(IS＜4.5) | 67 | 37 | 36.04 |  |  |  |  |  |  |  |
| High(IS≥4.5) | 62 | 24 | 39.75 | 0.66 | 0.40-1.10 | 0.114 |  | 0.68 | 0.39-1.16 | 0.158 |
| **Invasive depth** |  |  |  |  |  |  |  |  |  |  |
| T1-2 |  |  |  |  |  |  |  |  |  |  |
| Low(IS＜4.5) | 39 | 5 | 49.40 |  |  |  |  |  |  |  |
| High(IS≥4.5) | 44 | 6 | 52.63 | 0.62 | 0.19-2.05 | 0.433 |  | 0.82 | 0.22-2.98 | 0.758 |
| T3-4 |  |  |  |  |  |  |  |  |  |  |
| Low(IS＜4.5) | 90 | 40 | 39.13 |  |  |  |  |  |  |  |
| High(IS≥4.5) | 90 | 27 | 44.34 | 0.63 | 0.38-1.02 | 0.059 |  | 0.69 | 0.41-1.15 | 0.154 |
| **Lymph node metastasis** | |  |  |  |  |  |  |  |  |  |
| Positive |  |  |  |  |  |  |  |  |  |  |
| Low(IS＜4.5) | 66 | 36 | 36.55 |  |  |  |  |  |  |  |
| High(IS≥4.5) | 57 | 23 | 39.31 | 0.70 | 0.42-1.18 | 0.184 |  | 0.70 | 0.40-1.22 | 0.206 |
| Negative |  |  |  |  |  |  |  |  |  |  |
| Low(IS＜4.5) | 63 | 10 | 48.26 |  |  |  |  |  |  |  |
| High(IS≥4.5) | 77 | 9 | 52.31 | 0.69 | 0.28-1.70 | 0.417 |  | 0.69 | 0.25-1.87 | 0.463 |
| **Distant metastasis** | |  |  |  |  |  |  |  |  |  |
| Positive |  |  |  |  |  |  |  |  |  |  |
| Low(IS＜4.5) | 9 | 7 | 20.44 |  |  |  |  |  |  |  |
| High(IS≥4.5) | 11 | 7 | 27.18 | 0.58 | 0.19-1.74 | 0.330 |  | 0.71 | 0.19-2.72 | 0.618 |
| Negative |  |  |  |  |  |  |  |  |  |  |
| Low(IS＜4.5) | 120 | 25 | 43.66 |  |  |  |  |  |  |  |
| High(IS≥4.5) | 123 | 39 | 49.02 | 0.58 | 0.35-0.96 | **0.033** |  | 0.61 | 0.36-1.04 | 0.072 |
| **Tumor deposits** |  |  |  |  |  |  |  |  |  |  |
| Positive |  |  |  |  |  |  |  |  |  |  |
| Low(IS＜4.5) | 10 | 6 | 27.90 |  |  |  |  |  |  |  |
| High(IS≥4.5) | 19 | 10 | 28.47 | 0.89 | 0.32-2.45 | 0.823 |  | 1.22 | 0.43-3.47 | 0.711 |
| Negative |  |  |  |  |  |  |  |  |  |  |
| Low(IS＜4.5) | 88 | 29 | 41.34 |  |  |  |  |  |  |  |
| High(IS≥4.5) | 85 | 13 | 46.72 | 0.40 | 0.21-0.77 | **0.006** |  | 0.44 | 0.21-0.92 | **0.028** |
| **Perineural invasion** | |  |  |  |  |  |  |  |  |  |
| Positive |  |  |  |  |  |  |  |  |  |  |
| Low(IS＜4.5) | 74 | 29 | 38.46 |  |  |  |  |  |  |  |
| High(IS≥4.5) | 64 | 19 | 40.67 | 0.72 | 0.40-1.28 | 0.258 |  | 0.78 | 0.42-1.46 | 0.441 |
| Negative |  |  |  |  |  |  |  |  |  |  |
| Low(IS＜4.5) | 27 | 7 | 46.05 |  |  |  |  |  |  |  |
| High(IS≥4.5) | 40 | 4 | 48.40 | 0.31 | 0.09-1.08 | 0.065 |  | 0.36 | 0.09-1.48 | 0.157 |
| **Lymphatic/venous invasion** | | |  |  |  |  |  |  |  |  |
| Positive |  |  |  |  |  |  |  |  |  |  |
| Low(IS＜4.5) | 29 | 14 | 36.80 |  |  |  |  |  |  |  |
| High(IS≥4.5) | 32 | 10 | 39.17 | 0.66 | 0.29-1.50 | 0.320 |  | 0.94 | 0.38-2.33 | 0.899 |
| Negative |  |  |  |  |  |  |  |  |  |  |
| Low(IS＜4.5) | 100 | 32 | 43.70 |  |  |  |  |  |  |  |
| High(IS≥4.5) | 102 | 22 | 48.70 | 0.60 | 0.35-1.04 | 0.066 |  | 0.66 | 0.37-1.78 | 0.158 |
| **Growth pattern** |  |  |  |  |  |  |  |  |  |  |
| Infiltrative |  |  |  |  |  |  |  |  |  |  |
| Low(IS＜4.5) | 80 | 36 | 39.32 |  |  |  |  |  |  |  |
| High(IS≥4.5) | 70 | 22 | 43.34 | 0.67 | 0.39-1.14 | 0.136 |  | 0.78 | 0.45-1.35 | 0.372 |
| cloddy/nested |  |  |  |  |  |  |  |  |  |  |
| Low(IS＜4.5) | 49 | 10 | 47.14 |  |  |  |  |  |  |  |
| High(IS≥4.5) | 63 | 10 | 51.30 | 0.70 | 0.29-1.67 | 0.416 |  | 0.52 | 0.19-1.40 | 0.195 |
| **Differentiation degree** | |  |  |  |  |  |  |  |  |  |
| poor/mucinous |  |  |  |  |  |  |  |  |  |  |
| Low(IS＜4.5) | 42 | 21 | 34.69 |  |  |  |  |  |  |  |
| High(IS≥4.5) | 33 | 15 | 37.24 | 0.84 | 0.43-1.64 | 0.612 |  | 0.72 | 0.37-1.40 | 0.330 |
| well/moderate |  |  |  |  |  |  |  |  |  |  |
| Low(IS＜4.5) | 82 | 22 | 46.78 |  |  |  |  |  |  |  |
| High(IS≥4.5) | 94 | 14 | 51.21 | 0.52 | 0.27-1.02 | 0.058 |  | 0.68 | 0.34-1.35 | 0.271 |
| **Maximum diameter （cm）** | | |  |  |  |  |  |  |  |  |
| >4 |  |  |  |  |  |  |  |  |  |  |
| Low(IS＜4.5) | 58 | 27 | 37.37 |  |  |  |  |  |  |  |
| High(IS≥4.5) | 61 | 16 | 45.91 | 0.49 | 0.26-0.91 | **0.023** |  | 0.62 | 0.32-1.18 | 0.143 |
| ≤4 |  |  |  |  |  |  |  |  |  |  |
| Low(IS＜4.5) | 70 | 18 | 46.67 |  |  |  |  |  |  |  |
| High(IS≥4.5) | 73 | 16 | 48.29 | 0.83 | 0.42-1.63 | 0.589 |  | 0.82 | 0.39-1.70 | 0.586 |
| **Family history** |  |  |  |  |  |  |  |  |  |  |
| Positive |  |  |  |  |  |  |  |  |  |  |
| Low(IS＜4.5) | 27 | 9 | 41.23 |  |  |  |  |  |  |  |
| High(IS≥4.5) | 27 | 4 | 49.26 | 0.40 | 0.12-1.30 | 0.126 |  | 0.59 | 0.17-2.00 | 0.393 |
| Negative |  |  |  |  |  |  |  |  |  |  |
| Low(IS＜4.5) | 102 | 37 | 42.55 |  |  |  |  |  |  |  |
| High(IS≥4.5) | 107 | 28 | 46.88 | 0.68 | 0.42-1.11 | 0.122 |  | 0.69 | 0.41-1.16 | 0.161 |
| [**Chemotherapy**](javascript:void(0);) |  |  |  |  |  |  |  |  |  |  |
| Yes |  |  |  |  |  |  |  |  |  |  |
| Low(IS＜4.5) | 57 | 16 | 47.07 |  |  |  |  |  |  |  |
| High(IS≥4.5) | 49 | 11 | 48.64 | 0.77 | 0.36-1.67 | 0.511 |  | 0.73 | 0.35-1.68 | 0.514 |
| No |  |  |  |  |  |  |  |  |  |  |
| Low(IS＜4.5) | 48 | 18 | 40.26 |  |  |  |  |  |  |  |
| High(IS≥4.5) | 58 | 12 | 47.42 | 0.49 | 0.24-1.03 | 0.058 |  | 0.53 | 0.25-1.15 | 0.108 |
