# Supplementary material for: DNA repair protein XPA is differentially expressed in colorectal cancer and predicts better prognosis
Source: Cancer Med. 2018 Apr 19;7(6):2339–49. doi: 10.1002/cam4.1480 (PMC6010851; doi:10.1002/cam4.1480)
Supplement: Supplementary file 2 — Table S2. Correlation between XPA mRNA expression and survival in colon cancer and rectal cancer ( based on TCGA). [file CAM4-7-2339-s002.docx]

| **Correlation between XPA mRNA expression and survival in colon cancer ( based on TCGA)** | | | | | | |
| --- | --- | --- | --- | --- | --- | --- |
| **XPA expression** | **Cases** | **Cases of events** | **MST** |  | **Univariate** |  |
|  |  |  |  | **HR** | **95%CI** | **P** |
|  |  |  |  |  |  |  |
| Low | 238 | 41 | 100.90 |  |  |  |
| High | 238 | 62 | 83.55 | 0.84 | 0.57-1.25 | 0.398 |
| **Stratification** |  |  |  |  |  |  |
| **Age** |  |  |  |  |  |  |
| ＞60 |  |  |  |  |  |  |
| Low | 157 | 31 | 83.79 |  |  |  |
| High | 180 | 48 | 81.17 | 0.94 | 0.60-1.49 | 0.805 |
| ≤60 |  |  |  |  |  |  |
| Low | 81 | 10 | 114.80 |  |  |  |
| High | 58 | 14 | 91.81 | 0.67 | 0.29-1.51 | 0.331 |
| **Gender** |  |  |  |  |  |  |
| Male |  |  |  |  |  |  |
| Low | 128 | 17 | 83.17 |  |  |  |
| High | 124 | 39 | 72.54 | 0.59 | 0.33-1.06 | 0.078 |
| Female |  |  |  |  |  |  |
| Low | 110 | 24 | 100.18 |  |  |  |
| High | 114 | 23 | 96.21 | 1.28 | 0.72-2.26 | 0.407 |
| **TNM stage** |  |  |  |  |  |  |
| I-II |  |  |  |  |  |  |
| Low | 126 | 14 | 94.06 |  |  |  |
| High | 141 | 20 | 98.25 | 1.21 | 0.60-2.42 | 0.596 |
| III-IV |  |  |  |  |  |  |
| Low | 107 | 26 | 94.54 |  |  |  |
| High | 91 | 38 | 66.03 | 0.66 | 0.40-1.09 | 0.106 |

| **Correlation between XPA mRNA expression and survival in rectal cancer ( based on TCGA)** | | | | | | |
| --- | --- | --- | --- | --- | --- | --- |
| **XPA expression** | **Cases** | **Cases of events** | **MST** |  | **Univariate** |  |
|  |  |  |  | **HR** | **95%CI** | **P** |
|  |  |  |  |  |  |  |
| Low | 83 | 10 | 66.74 |  |  |  |
| High | 82 | 16 | 79.87 | 0.81 | 0.36-1.78 | 0.591 |
| **Stratification** |  |  |  |  |  |  |
| **Age** |  |  |  |  |  |  |
| ＞60 |  |  |  |  |  |  |
| Low | 59 | 10 | 47.17 |  |  |  |
| High | 49 | 12 | 67.07 | 0.96 | 0.41-2.25 | 0.925 |
| ≤60 |  |  |  |  |  |  |
| Low | 24 | 1 | 52.04 |  |  |  |
| High | 33 | 4 | 57.77 | 0.02 | 0.00-85.14 | 0.360 |
| **Gender** |  |  |  |  |  |  |
| Male |  |  |  |  |  |  |
| Low | 44 | 5 | 71.59 |  |  |  |
| High | 46 | 8 | 78.67 | 1.02 | 0.33-3.14 | 0.969 |
| Female |  |  |  |  |  |  |
| Low | 39 | 5 | 52.7 |  |  |  |
| High | 36 | 8 | 78.59 | 0.65 | 0.21-2.03 | 0.458 |
| **TNM stage** |  |  |  |  |  |  |
| I-II |  |  |  |  |  |  |
| Low | 48 | 5 | 76.52 |  |  |  |
| High | 33 | 1 | 123.26 | 0.67 | 0.22-2.10 | 0.497 |
| III-IV |  |  |  |  |  |  |
| Low | 31 | 4 | 48.73 |  |  |  |
| High | 44 | 12 | 61.71 | 3.82 | 0.45-32.75 | 0.221 |

MST, median survival time;
